# The endangered brain: actively preserving *ex-situ* animal behaviour and cognition will benefit *in-situ* conservation

**DOI:** 10.1098/rsos.230707

**Published:** 2023-08-30

**Authors:** Fay E. Clark, Alison L. Greggor, Stephen H. Montgomery, Joshua M. Plotnik

**Affiliations:** ^1^ School of Psychological Science, University of Bristol, Bristol, UK; ^2^ School of Biological Science, University of Bristol, Bristol, UK; ^3^ San Diego Zoo Wildlife Alliance, Escondido, CA, USA; ^4^ Department of Psychology, Hunter College, City University of New York, New York, NY, USA; ^5^ Department of Psychology, The Graduate Center, City University of New York, New York, NY, USA

**Keywords:** animal behaviour, cognition, enrichment, neuroscience, reintroduction, zoo

## Abstract

Endangered species have small, unsustainable population sizes that are geographically or genetically restricted. *Ex-situ* conservation programmes are therefore faced with the challenge of breeding sufficiently sized, genetically diverse populations earmarked for reintroduction that have the behavioural skills to survive and breed in the wild. Yet, maintaining historically beneficial behaviours may be insufficient, as research continues to suggest that certain cognitive-behavioural skills and flexibility are necessary to cope with human-induced rapid environmental change (HIREC). This paper begins by reviewing interdisciplinary studies on the ‘captivity effect’ in laboratory, farmed, domesticated and feral vertebrates and finds that captivity imposes rapid yet often reversible changes to the brain, cognition and behaviour. However, research on this effect in *ex-situ* conservation sites is lacking. This paper reveals an apparent mismatch between *ex-situ* enrichment aims and the cognitive-behavioural skills possessed by animals currently coping with HIREC. After synthesizing literature across neuroscience, behavioural biology, comparative cognition and field conservation, it seems that *ex-situ* endangered species deemed for reintroduction may have better chances of coping with HIREC if their natural cognition and behavioural repertoires are actively preserved. Evaluating the effects of environmental challenges rather than captivity *per se* is recommended, in addition to using targeted cognitive enrichment.

## 1. Introduction

We are currently experiencing the planet's sixth mass extinction [1]. The United Nations established 20 ‘AICHI’ biodiversity targets' in 2010 to address and mitigate rapid biodiversity loss across the globe [2]; we failed to meet most targets by 2020 including the target to prevent species extinction [3,4]. As this environmental crisis intensifies, conservation programmes are under increasing pressure to justify actions to governments, funders, the public and other stakeholders [5]. Among the most scrutinized conservation actions is *ex-situ* conservation (as defined in table 1).

**Table 1.** Glossary of terms.

term	definition
captivity	All settings where animals have confined housing and/or are under human management.
captivity effect	The effect(s) of living in captivity on the brain, body and behaviour of animals.
challenge	An environmental situation that pushes an animal above its own baseline, to engage or develop evolved cognitive skills.
cognition	The mental processes by which animals collect, retain and use information from the environment to guide their behaviour [6].
cognitive enrichment	Enrichment that specifically aims to challenge evolved cognitive skill(s) to either enhance future cognitive skill, or welfare.
cognitive or behavioural flexibility	The ability to effectively change behaviour in response to changing environmental conditions [7].
conservation breeding	The action of creating and maintaining sustainable populations of animals *ex-situ*, through reproductive and genetic management.
domestication	Adaptations to captivity that arise from artificial selection by humans for certain behavioural, morphological and genetic traits.
endangered	At a very high threat of extinction (species level). Endangered species have small, unsustainable population sizes that are geographically or genetically restricted [8].
enrichment	A purposeful addition of challenge to the captive environment to modify cognition, behaviour or welfare state [9,10].
*ex-situ* site	A captive site where animals are managed for conservation purposes. Includes: zoos, safari parks, aquariums, wildlife sanctuaries, research centres, and temporary field stations within range countries.
Human-induced rapid environmental change (HIREC)	The phenomenon where the wild environment is rapidly altered by human activities and their outcomes. HIREC exposes animals to novel selection pressures that are vastly different to the ones they have evolved to overcome [11].
neuroplasticity	The ability of the brain's structure/function to change as a result of life experience.
reintroduction	The intentional movement and release of an organism inside its indigenous range from which it has disappeared [12, p. 2].

The International Union for Conservation of Nature (IUCN) classifies species by their level of risk of extinction, and these classifications are used globally for species conservation. Vulnerable, endangered and critically endangered species are considered to be threatened with extinction [8]. These threatened categories have criteria describing the species’ population size/s and geographical range/s. Currently, around 20% of all assessed vertebrates are threatened with extinction, and conservation breeding programmes are a growing strategy to halt extinction [13]. In line with IUCN categorization, *ex-situ* conservation programmes have focused on maximizing the total numbers, and genetic diversity, of individuals bred in *ex-situ* sites, via species survival plans [14–16]. *Ex-situ* sites are therefore commonly referred to as metaphorical arks or safety nets against extinction [17,18].

However, the physical body of an animal is not the only thing at risk of extinction; animal cognition and behaviour, and the neural substrates underlying them, are also at risk (at least of permanent maladaptive change). Yet this ‘endangered brain’ concept is a paradox. Conservation breeding aims to produce individuals that survive and reproduce, but living in captivity can significantly hinder the development and expression of cognitive and behavioural skills required for survival, therefore putting the species at further risk of extinction. This paper reviews evidence that captivity is linked to cognitive/behavioural loss or modification, and that certain changes can detrimentally impact animal survival [e.g. 19–21]. Most evidence for the captivity effect comes from laboratory and farmed animals, in addition to a handful of historical zoo specimens (particularly carnivores) with no accompanying cognitive or behavioural records [21]. This is concerning, given that approximately 15% of threatened species are housed in zoos [22]. Furthermore, cognition rarely features in conservation action plans [23].

A recently published review of phenotypic effects of captivity [21] gave significantly more focus to physical and physiological health than cognition. To address this gap in the literature, the current paper focuses primarily on what we know about the vertebrate brain in a captive state, synthesizing across multiple disciplines: neuroscience, sensory biology, behavioural biology and comparative cognition (§2). It then considers the vertebrate brain in its wild state under human-induced rapid environmental change (HIREC; §3), before considering the challenged brain, i.e. the extent to which enrichment has been used in *ex-situ* sites for reintroduction purposes, and more widely in captivity (§4). Finally, we propose a new framework to evaluate environments by the level of challenge they provide, rather than a captive/wild dichotomy or placing laboratories, farms, zoos, etc. into artificial siloes (§5).

This paper does not evaluate the ethics or success of the existing practice of conservation breeding. Instead, readers should refer to a number of comprehensive reviews spanning the last five decades including high-profile success stories such as the Przewalski's horse (*Equus ferus przewalskii*) and black-footed ferret (*Mustela nigripes*) [21,24–26]. Furthermore, the paper does not address how the *in-situ* geographical ranges or genetic pools of various species became threatened in the first place, in other words, we do not review the causes of human-induced rapid environmental change (see [1–3,11]). Species conservation has always been a holistic venture with practitioners working collaboratively *in-situ* and *ex-situ* [25,27,28]. So, the focus of this paper is to question whether individuals housed in *ex-situ* programmes have the mental and behavioural tools necessary for ongoing survival, a topic that has been relatively overshadowed by other collaborative efforts.

## 2. The captive brain

### 2.1. A primer on the brain

In vertebrates, many studies have sought to link whole or partial brain size (volume or mass) to a range of characteristics including social structure, foraging style or generalized intelligence (for example, more than 50 cross-species analyses were performed prior to 2006 [29]). Relative brain size, which corrects for animal body size, is often seen as particularly important because larger animals tend to have larger brains, and vice versa [30], although some authors argue absolute size may more directly reflect functional performance [31]. The relative size of the whole brain is also often taken as a global measure of animal cognitive ability, but the relative measurement of brain areas responsible for specific cognitive functions is arguably more valid [30]. In dead yet preserved animal specimens, the brain can be removed and weighed to estimate its total size [e.g. 32], and dissected or imaged to reveal the relative size of specific brain regions [e.g. 33–35]. In skeletal specimens where the brain has already decayed, the volume of the remaining braincase of the skull can be measured by pouring water, sand or other fine-grained material into this shape. In live specimens, brain data can be obtained by placing sedated animals into an MRI scanner [36] or, in a hybrid approach, post-mortem brains may be scanned to reveal internal brain structure and an estimate of brain volume [37].

Due to space limitations, more thorough critiques of what brain size means in terms of animal cognition and behaviour will not be covered here [instead see 29,30]. In this paper, whole brain size is discussed primarily as the most accessible source of data, under the assumption that any within-species changes observed in whole brain size may also reflect specific changes in composition.

### 2.2. Summary of the captivity effect

**Figure 1.**
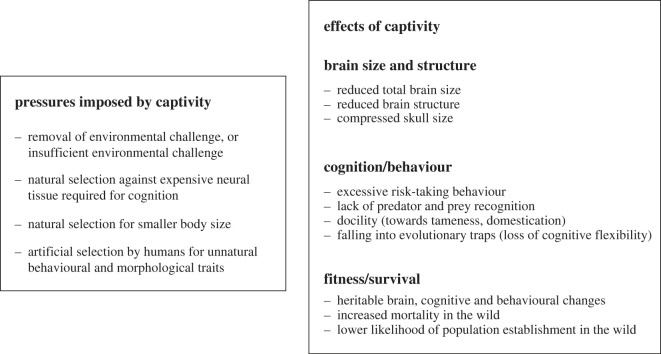
An overview of the captivity effect in vertebrates.

Current evidence for the captivity effect (figure 1.) mostly stems from data from animals housed in laboratories and farms (including fisheries) as well as domesticated and feral species (see also a recent review focusing on morphological changes [21]). The question of why evidence is lacking for a captivity effect in animals in *ex-situ* conservation sites is important because the absence of evidence does not mean the effect does not exist. Endangered species are by definition rare, and where they exist, small *ex-situ* sample sizes [38] are not conducive to the large-scale comparative neural research normally undertaken on laboratory model animals. Animal lifespan can also be extended in captivity versus the wild [38] so it may take a long time to access brain and other morphological materials at post-mortem from species with slow life histories. Another significant obstacle is being able to sedate large, dangerous or anaesthetic-sensitive animals for live brain scanning; this is not feasible for many if not most *ex-situ* sites.

### 2.3. Brain size and structure

The size of the total brain or its various structures provides insight into how captivity impacts animals at a gross neurological level. For example, captive Trinidadian guppies (*Poecilia reticulata*) and steelhead trout (*Oncorhynchus mykiss*) have smaller brains than their wild counterparts [34,35], and variation in brain size is also linked to survival within captive guppy populations [39]. However, this is not always the case. Brain volume is not reduced in stripe-faced dunnarts (*Sminthopsis macroura*) reared in captivity for 2–7 generations compared with wild specimens [40] (although records on this research colony suggest there was a high level of enrichment).

Effects of long-term captivity on the brain are most dramatically illustrated in domesticated species. In general, domesticated animals have smaller brains than their wild ancestors [e.g. cattle, chicken, Mongolian gerbils (*Meriones unguiculatus*) 32,33,41] and some specific brain structures, including the telencephalon and cerebellum, are linked to increased fearfulness and aggression [33,42,43] or increased levels of human contact [41]. Some typical morphological traits in domesticated mammals like ‘flat-facedness’ are linked to abnormal embryonic cell development rather than brain size [44,45]. Brain changes in captivity could also be linked to the high energetic cost of neural tissue [45,46], which may lead to rapid degradation of neural traits (or behaviours) that no longer contribute to survival [46]. Studies in domesticated animals reveal that changes in brain size and structure can occur over relatively short time periods. For example, in the Russian farm-fox experiment artificially recreating an accelerated wolf-to-dog domestication, silver foxes (*Vulpes vulpes*) selected for tameness and aggression showed significant changes to the prefrontal cortex and hypothalamus in less than 100 generations [43]. The loss of genetic diversity can also be rapid, adding further strain to the maintenance of natural phenotypic diversity [47–49].

Domesticated species are likely under more targeted selection than animals in *ex-situ* conservation sites; nevertheless, the studies discussed above raise the prospect of substantial neural changes in captive populations over short time periods. At a shorter timescale, feral populations of domesticated species may provide some clues as an extreme analogy for prolonged captivity [50,51]. Interestingly, feralization does not result in a simple reversal in trends of brain size reduction and composition; feral populations display small differences in brain size compared to domesticated species, and potential changes in brain structure that differ from both wild and domesticated species [51]. Genomic findings on feral chickens are consistent with this non-reversal effect, showing genes targeted by selection during domestication and feralization are independent [52].

Very little data are available on vertebrate brain size and structure in *ex-situ* populations, compared with laboratory, farmed and domesticated species. However, there is some evidence of sizeable differences in brain/skull size between wild animals and their captive counterparts where captivity has been maintained for a few decades or less. A reduction in black-footed ferret skull size is detected after less than 10 years of captive breeding; captive skulls are 5–10% smaller than wild skulls and genetic analyses rule out simple effects of inbreeding [53]. There could be selection pressure for a smaller body size in captivity that would rebound back to wild levels if the animals were reintroduced, but this lacks empirical investigation. Indeed, in feral populations of domestic pigs, cranial capacity does not rebound to its ancestral levels [54]. Similar patterns are also observed in species currently under threat. For example, cranial volume (a proxy for brain size) of lions *Panthera leo* and tigers *P. tigris* is reduced in *ex-situ* specimens compared to wild specimens [55]. Similarly, absolute and relative brain volume across 21 species of Anseriforme waterfowl in captive breeding programmes are reduced versus wild specimens [56].

Carnivores are particularly vulnerable to extinction and have therefore been subject to intense conservation breeding, but also appear particularly vulnerable to morphological changes in captivity [57]. Deformations in skull morphology have been found to reduce space for, and possibly compress, particular brain structures in lions [58]. These effects may also be compounded by ecology. Among canid species, those with more specialized carnivorous diets have the most pronounced differences in cranial shape between wild and captive samples [59]. However, metanalyses suggest the effect of captivity on cranial morphology can be varied and unpredictable across species. This highlights the need for further comparative research and more consistent methodologies to measure variation in brain structure [30].

Changes to adult brain size and structure observed in a very small selection of zoo-housed vertebrates thus far could be due to genetic effects in these populations accumulated over multiple generations. This would be consistent with wider evidence of heritable variation of both brain morphology [36,60] and behavioural traits [61,62] in captive mammals. However, substantial differences in brain size and structure can also be produced over more immediate time scales due to neuroplasticity during development. Postnatal brain growth is substantial in some mammalian species and can correlate with ecological traits [63,64]. Brain growth is also indeterminate in some species, thus extending the window for the environment to shape its development. For example, brain development (and consequently behaviour) can be impacted by a range of stimuli in mammals and fish including sensory cues [65], social interactions [66,67], predator threats [66], and locomotor experience [67]. In sticklebacks (*Pungitius pungitius*) short-term changes in brain structure can be induced by environmental changes and can impact behavioural decision-making, including social interactions [65], which could then have broader impacts on population dynamics.

Thus far, research on the captivity effect has focused on the size of the whole brain, or partial brain regions, and reports a reduction in brain size as a negative (undesirable) result. To be a concern for conservation practice, this brain change must be linked to fitness or survival in natural populations. Indeed, a study across 236 bird species found that species with larger brains (relative to their body size) have lower mortality, supporting the cognitive buffer hypothesis of large brain evolution [68], and in experimental populations of fish, brain size can aid survival from predation [39]. Numerous other studies point towards the survival benefits of larger overall brain size, such as more effective population establishment in mammals, reptiles and amphibians [69,70]. These comparative analyses often assume these effects are due to cognitive or behavioural flexibility [71], again highlighting the importance of plasticity and environmental effects on behaviour. However, how these effects play out within rather than between species is not yet clear. It is also important to acknowledge that other morphological changes can affect survival (for example wing shape in orange-bellied parrots, *Neophema chrysogaster*; [72]) making it difficult to isolate the effect of morphological changes to the brain from morphological changes to the body.

### 2.4. Cognition and behaviour

Long or short-term changes to animal brain size or structure could be linked to the expense of brain tissue, and therefore the rapid loss of any brain structures that do not contribute to fitness [46,65]. Alternatively, brain changes can result from artificial selection by humans for behavioural syndromes or temperaments such as docility that make animals easier to work with or manage [73,74]. Even though animals living in *ex-situ* sites may not purposely be subjected to this type of selective breeding for behavioural traits, breeding can be inadvertently biased towards behavioural phenotypes that better cope with the *ex-situ* environment and human caregivers can exert unconscious selection in choosing breeding pairings [74].

There is some evidence for a link between changes in brain size/structure and cognitive ability and behavioural repertoire across and within species both in the wild and captivity (across mammals, birds; [75,76] within birds, fish [77,78]). For example, the performance of stereotypic behaviour (a repetitive, invariant behaviour pattern with no obvious goal or function [79]) has been speculatively linked to changes in cranial morphology in large terrestrial mammals (Asian rhinoceros *Rhinoceros* and *Dicerorhinus* spp. [80], tigers [81] and pigs *Sus scrofa* [82]), but without a clear cause-effect relationship. It is conceivable that stereotypical behaviour leads to changes in muscle usage which then leads to morphological changes [59,82], rather than morphological changes leading to pathological behaviour. Interestingly, there is no significant morphological drift seen in older versus newer post-mortem specimens collected from *ex-situ* sites [59]. This could suggest the experience of captivity has changed little over time and it is always going to be inherently different to that of the wild. Alternatively, any improvements in the captive experience (i.e. due to changes to animal housing and husbandry) might be outweighed by other negative effects such as a response to artificial selection regimes imposed by multiple generations of captivity and inbreeding.

Regardless of their aetiology, stereotypical behaviours might have negative implications for reintroduction because they take the place of other more desirable behaviours in the animal's repertoire. For example, stereotypical behaviours have been associated with decreased behavioural flexibility and engagement with the environment [83]. There seem to be no reports of stereotypic behaviour extinguishing following an animal's reintroduction; stereotypical behaviour is more likely to remain a scar of earlier life experience, as supported by its perpetuation in captive animals placed into different environments [84,85]. The effects of prolonged captivity on the dampening of other behavioural traits such as predator avoidance and reproductive behaviour have also been documented [86,87], some of which are unlikely to be regained without intervention [88]. Since the erosion of these traits in human care is not a given [89], more research from *ex-situ* sites would allow for better predictions on the likelihood of behavioural impacts. The link between animal cognition and affective state (i.e. short-term emotions and long-term moods) also has relevance here. The Affect as Information hypothesis [90] states that current affective state impacts cognition, namely the ability to make judgements. This is supported in a number of nonhuman primate species; for example, captive Guinea baboon (*Papio papio*) response times on a computerized cognitive task are slower when they have a negative mood, versus a neutral or positive mood [91]. Captivity-induced depression [92] thus has the potential to compromise cognitive performance.

### 2.5. Fitness and survival

At this juncture, one may be wondering whether endangered species released from *ex-situ* sites have poorer fitness and survival outcomes than wild-to-wild translocated animals *in-situ*. In other words, what do we know about the effect of captivity on the post-release success of threatened species? The topic of animal reintroduction (and more widely animal translocation which covers any human-mediated movement for conservation benefit [12]) is vast and spans several decades (for review see [12,93–95]). While *ex-situ* sites have played a critical role in saving numerous vertebrate species that were previously extinct in the wild [22,27], reintroduction of captive vertebrates has sometimes been criticized for low overall success (e.g. success rate from wild sources 31% versus *ex-situ* sources 13% [96]), although it should be noted this reference is now over 20 years old.

Recent data, however, paint a more positive view. A 2018 IUCN report containing 42 vertebrate case studies classified 73% of reintroductions (some reintroductions to multiple sites) as successful or highly successful, although the definitions of success varied widely [97]. And while more animals are translocated wild-to-wild than from *ex-situ* sites (57% versus 23% respectively) there is a comparable success rate between captive and wild sources (around 88%) [98]. A systematic review of 514 terrestrial vertebrate translocations found that translocations of animals from *ex-situ* sites were marginally more successful than those of animals from the wild (76% versus 70%, respectively). However, animals reintroduced from *ex-situ* sites were more likely to have a declining population growth rate [93] than those moved from elsewhere in the wild. Further analysis focusing on vertebrate reintroductions from *ex-situ* sites, with a categorization of the nature of captivity (e.g. duration, housing type, but see §5 for an alternative classification) is required to fully understand the effect of captivity on reintroduction. Thus far, there is some evidence from reptiles that captive-rearing duration may be more important than environmental enrichment for survival [99,100]. For a new empirical analysis to be of real benefit, it must address the prevailing issue of how to define reintroduction successes and failures in an operational manner [101]. It would be helpful to expand the definition of reintroduction success to consider animal welfare, i.e. any stress, pain or suffering related to reintroduction, which has been a topic overshadowed by focusing on maximum population sizes and ranges [102].

In lieu of specific data linking a captivity effect with endangered species reintroduction failure, it is necessary to try and extrapolate from non-endangered species in laboratories and farmed fish. In fish, there is mounting evidence that the long-term genetic effects of captivity can affect population survival in the wild (steelhead trout [103]), and that captive conditions can even reduce survival in a single generation (Atlantic salmon, *Salmo salar* [104]), but the specific contribution of captivity-induced changes in brain development to these survival deficits is difficult to quantify. While there is evidence that intraspecific variation in fish brain size can affect survival in semi-natural conditions containing natural predators [39], perhaps the best evidence for a link between the captive environment and the probability of survival in the wild comes from farmed or endangered fish, where individuals are released from hatcheries to supplement wild populations. Here, genetic adaptation to captivity can occur quickly [105] and captive breeding can cause reduced fitness in the wild [104], leading to negative impacts on population recovery rates if genetic changes occur in captive animals later released into the wild [106]. These kinds of effects could explain why population growth is higher when the source population comes from the wild rather than captivity [12].

## 3. The wild brain

A persistent debate in the literature is whether the wild is justifiably a ‘better’ site for animals to be conserved than *ex-situ* and vice versa [5,17,18,107]. Fundamental ethical standpoints on animal freedom withstanding, it is increasingly recognized that HIREC poses significant survival risks to wild animals, and without these animals, ecosystem processes can degrade [11,108].

### 3.1. Environmental change and traps

While the brains of wild animals have been shaped by relatively predictable threats encountered over their long evolutionary histories, wild animals are now being challenged at unprecedented levels by HIREC [11,109,110]. Five major categories of HIREC are recognized: habitat loss and fragmentation, environmental pollution, climate change, over-harvesting and the spread of exotic species [11]. These categories can also overlap; for example, habitat loss can contribute to climate change, and therefore HIREC should be considered as a suite of connected issues [11]. HIREC is responsible for introducing novel threats that many animals are simply not adapted to overcome. Many anthropogenic environmental stimuli may fail to activate adaptive behavioural responses due to the novelty or unpredictability of the stimuli and thus affect the likelihood of survival [110–113]. The strong selective pressures humans exert upon animal behaviour and its underlying cognitive processes are clear in the case of ecological or evolutionary traps (ecological traps are habitat-based whereas evolutionary traps are at a wider scale) [113]. Animals are ‘trapped’ when their natural response to the environment is no longer associated with expected survival outcomes. These traps can lead to issues across the cognitive domains of perception, learning, memory and decision-making [20] such as maladaptive habitat preferences [114] or the mis-categorization of food or predators [115,116]. For example, marine turtles (Family Cheloniidae) and mosquitofish (*Gambusia affinis*) can mistake marine plastics for their normal diets because their evolved search images fail to distinguish these threats from their prey [117,118]. HIREC also pushes animals into conflict involving humans; for example, brown bears (*Ursus arctos*) compete with humans over access to high-quality habitat, leading to increased bear mortality through hunting and vehicle strikes [119].

HIREC can disrupt other cognitive mechanisms too. For instance, fragmented habitats and reduced opportunities for conspecific interactions can disrupt social learning [120]. Reductions in population size can create Allee effects, leading to, for example, a breakdown in the expression of group behaviour such as lekking and communal defence [121], which can impact the potential for population recovery post-translocation [122]. For example, in the kakapo (*Strigops habroptilus*), a lek-breeding parrot, the Allee effect has been implicated in the remaining population having little possibility of recovery. Meanwhile, human-mediated removal of cues or experiences, such as the removal of top predators, can lead to the erosion of targeted perception and behaviour, such as predatory wariness and anti-predator responses [123].

### 3.2. Environmental change and cognitive flexibility

Most studies examining the impact of HIREC on vertebrates report negative outcomes [11,124,125]. Positive outcomes are infrequent, but when they do occur they are linked to a species having a high level of cognitive flexibility [20,126]. As a reminder, cognitive or behavioural flexibility is defined as the ability to effectively change behaviour in response to changing environmental conditions [7]. Urbanization as part of HIREC can provide animals with more widespread and predictable foraging and nesting opportunities, protection from predators and more stable microclimates [127]. Beyond cognitive flexibility, a number of allied behavioural or cognitive traits have been implicated in the success of ‘urban adaptor’ species, including neophilia, boldness, innovation, social learning and the ability to categorize humans and interpret their cues for danger [126,128–130]. Because the brain gives rise to cognition and behaviour, cognitive flexibility is linked with neuroplasticity (§2.2, [7]). Relatively large-brained animals have a higher propensity to innovate and learn, i.e. they have better cognitive flexibility (birds: [131], primates: [132]) and such flexibility helps them face challenges presented by new or altered environments [133,134]. However, while the ability of birds to innovate new behavioural solutions is linked to lower extinction risk [135], it has alternatively been shown to have no significant effect [136].

### 3.3. Cognition and conservation

The survival potential of species may be related to their overall brain size or cognitive or behavioural flexibility and allied skills such as the novelty response and ability to categorize. Cognitive or behavioural flexibility therefore appears to be a well-justified aim for *ex-situ* animals earmarked for reintroduction. So far, there is scant research in the area of captive-wild cognitive comparisons, and results have been mixed and not focused on endangered species. For example, a study of problem-solving in wild and captive hyenas (*Crocuta crocuta*) showed that captive animals were better problem-solvers, possibly due to less neophobia and higher exploration [137]. By contrast, wild and captive passerine birds show similar cognitive task performance [138]. Wild Goffin's cockatoos (*Cacatua goffiniana*) have comparable innovation skills to their captive counterparts, but a lower level of motivation [139]. These studies provide some evidence that captive birds can retain their wild-like skills under certain environmental conditions but offer little insight into the level of variation one might expect across different environments, thus calling for increased replication. One recent pair of studies in an endangered species, the Asian elephant (*Elephas maximus*), showed that both captive [140] and wild [141] elephants can innovate on a similar problem-solving task, although the authors did not compare performance between the populations. It is also likely that cognitive flexibility varies within species and within populations [112,141], suggesting that considering individual variation in certain cognitive traits, and its environmental or genetic determinants, should also be an important factor when considering how best to select *ex-situ individuals* for reintroduction.

## 4. The challenged brain

We define ‘challenge’ as an environmental situation that pushes an animal above its own baseline, to engage or develop evolved cognitive skills (table 1). The brains of animals are challenged by natural or man-made changes to the environment related to finding food, mates or other resources or avoiding threats [142]. Enrichment refers to challenges purposely added to captive environments [9,10] and is often used to simulate a wild environment [e.g. 143], but can also be highly functional and artificial [e.g. 144]. The primary aim of enrichment is usually to enhance animal welfare (e.g. zoos, farmed livestock and laboratory animals [10]) but it can also be used as an intervention to modify brain, cognitive and behavioural development and expression [7,10,145,146]. This paper focuses on two overarching types of enrichment with relevance to *ex-situ* conservation. First, general environmental enhancements can improve naturalistic brain development and promote naturalistic behaviours. Second, specific and targeted challenges or experiences prior to wild release can promote specific cognitive and behavioural skills thought to enhance survival [147–149].

### 4.1. Environmental enhancements

Given that captive rearing can significantly impact brain development (§2), enriched rearing environments have been used to promote behavioural competence and flexibility in laboratory rodents [150]. Low survival in salmon reintroduced from a hatchery setting is linked to excessive risk-taking behaviours, but when tanks are enriched with naturalistic vegetation, prey and predators, risky behaviours significantly decrease within two weeks [143]. However, in environmental enhancement studies such as this, it is not possible to parse the relative effect of habitat, prey and predator opportunity.

The positive effects of enrichment potentially extend beyond one generation, as the parental condition can also impact offspring brain size [151], neural gene regulation [152] and behaviour [153]. For example, maternal stress has been shown to alter offspring activity and feeding success in farmed salmon [154], while enrichment of the parental environment can alter maternal care [155,156] with subsequent effects on play behaviour in laboratory rodents [156]. Hence, by impacting behavioural development, these environmental effects can ripple through multiple generations [146], potentially influencing behavioural strategies expressed in the wild and the survival of reintroduced populations. This also means that *ex-situ* populations may require breeding multiple generations in semi-natural conditions before release.

### 4.2. Targeted challenges

Conservation programmes have used more specific enrichment as a tool to support the reintroduction of captive adult animals [147,157]. In zoos, there has been an overwhelming focus on environmental enrichment that recreates naturalistic-looking environments but is also motivated by giving aesthetic and educational value to visitors [10,158–160]. From the published literature, it seems conservation-focused enrichment has a number of goals including increasing behavioural diversity and skill learning [143,147–149,161]. A recent meta-analysis of 41 vertebrate translocations found that enrichment is associated with higher survival [162], but the authors did not identify which element(s) of enrichment are linked to survival success. The authors also viewed anti-predator training as separate to, not a subset of, enrichment (in contrast to [143]).

Enrichment in the form of skill learning can be achieved either through passive opportunities and social learning from conspecifics, or more active periods of training by humans [94]. Post-release survival in zoo-housed honeyeater birds (*Anthochaera phrygia*) was linked to pre-release song learning [161], and while it was not classified as a form of enrichment by the authors, the addition of song stimuli into the existing environment could be classified as such [10]. Other learning experiences may involve exposure to prey (black-footed ferrets [163]), adding flexibly moving vegetation from the wild habitat (Golden lion tamarins, *Leontopithecus rosalia* [164]), and puppet-rearing chicks to avoid human imprinting [165]. Predator and prey recognition training has been undertaken for mammals, birds, amphibians and fish (reviewed by [19,113,126]), along with training to hunt [166]. Anti-predator training has had mixed success and is highly dependent on species, specific method and release context, but a thorough evaluation is hindered by research focusing on measuring behavioural responses to training rather than post-release survival [19,113]. The ‘ecology of fear’ and more broadly the ‘landscape of fear’ conceptual frameworks acknowledge there are numerous behavioural and physiological costs and benefits to being fearful of predators, and success against predation is not simply defined as the ability to escape a discrete hunting episode [167,168].

**Figure 2.**
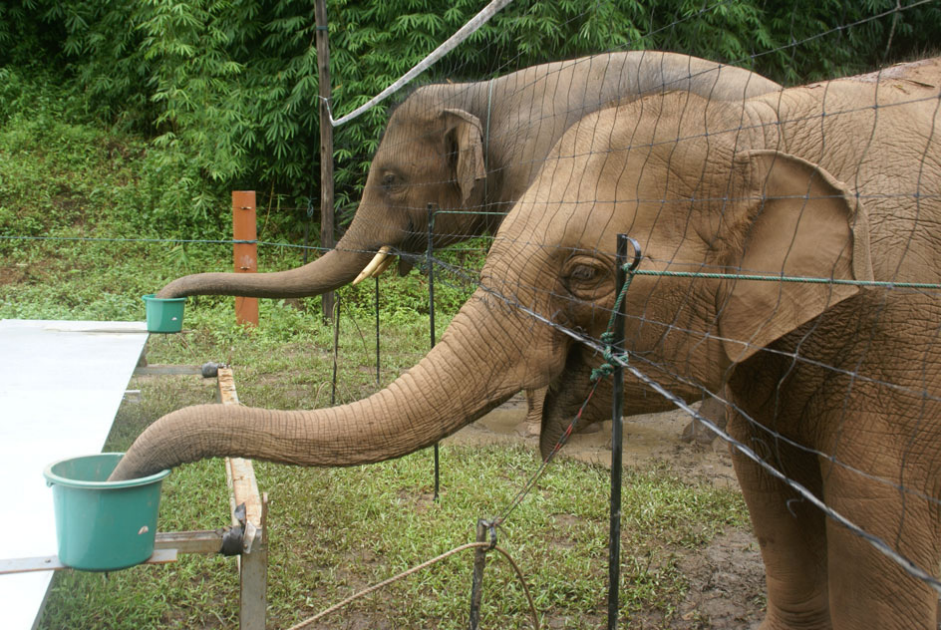
Two Asian elephants eat together after pulling ropes to gain access to an out-of-reach table. This is a classic cognitive test of cooperation. Image: J.M. Plotnik.

Even though targeted enrichment challenges have been used in *ex-situ* conservation programmes focused on reintroduction, the actual goal of enrichment varies and has lacked clear definition across the literature. Exposure to a prey species, for example, could challenge many cognitive domains or skills including memory, reasoning, problem-solving, spatial navigation and motor coordination. Enrichment that focuses on challenging a particular cognitive skill or cognitive performance in a given domain [6] has only recently begun to gain traction, broadly termed ‘cognitive enrichment’ [169] or ‘cognitive training’ [170]. Cognitive enrichment for conservation may therefore actually take the same physical form as cognitive tasks used in pure research; for example, puzzle boxes, tubes, string-pulling tasks or mazes [171,172] (figure 2).

## 5. Preserving the endangered brain

### 5.1. Challenge appraisal

This paper reviews how captivity can lead to changes in the brain, cognition and behaviour of vertebrates. But these changes come down to the type of challenges animals face, rather than being in captivity *per se*. In fact, challenges can vary considerably within one type of captive setting such as laboratories, and enrichment is an experimentally induced challenge that can be added to any type of captive setting. It has also been proposed that some aspects of captivity are broadly equivalent to some aspects of HIREC (e.g. habitat loss in the wild is equivalent to restricted movement in enclosures [173,174]), displacing the idea that a particular setting is ‘better’ for animals to reside. This leads to an important forward-thinking question: is a captive brain/wild brain dichotomy useful when thinking about the *ex-situ* conservation of endangered species? Rather than delineate different types of *ex-situ* site (zoos versus field stations, etc.), it is useful to appraise the type of challenges animals face relative to their evolved behavioural and cognitive skills and current HIREC conditions (table 2).

**Table 2.** A challenge appraisal for a fictitious endangered species, currently living in an *ex-situ* site, deemed for reintroduction. In this example, the site climate and social grouping are inadequate challenges, but exposure to anthropogenic noise and aerial threats may prepare animals for reintroduction.

*ex-situ* challenge	comparison to *in-situ* challenge	challenge appraisal
The enclosure temperature and humidity are on a thermostat and kept constant.	The climate is unstable and harsh. There are hot-dry and warm-damp seasons.	The *ex-situ* climatic challenge is inadequate because the species has evolved to cope with seasonal fluctuations. The animal may find it difficult to deal with increasingly harsh temperature fluctuations caused by HIREC when reintroduced.
The enclosure houses five individuals.	Group size ranges from 10–60, average 40. Social groups periodically undergo fission-fusion to forage in patchy habitat.	The *ex-situ* group size is too low; animals currently do not experience adequate challenges including food competition and mate choice.
The site is in a quiet countryside area. Animals receive regular exposure to traffic noise playback through speakers.	Due to HIREC, the species' territory defence call is masked by road traffic noise.	Research confirms *ex-situ* animals change the frequency and duration of territory calls in response to playback. This helps them to adapt to current HIREC conditions.
Commercial aircraft fly over the enclosure 10–20 times per week.	The main predator is a large bird of prey; the species responds by alarm-calling and hiding in vegetation.	Sporadic exposure to aircraft may simulate the threat of aerial predators, but research is needed to examine if the animals' response is appropriate (e.g. alarm-calling, hiding). Consider using more realistic aerial predator models and whether too regular and predictable challenge leads to habituation.

It is important to proceed with caution when considering the welfare outcomes of challenge. Challenges are more likely to be associated with overall good welfare outcomes if they can ultimately be overcome because the animal has the requisite cognitive or behavioural skills [142,169]. However, these challenges may still be associated with some temporary negative outcomes like frustration or distress. In the example provided (table 2), aerial predator exposure may be associated with brief distress upon initial predator exposure, but gradually lead to more adaptive behavioural responses and less fear. This being said, it is also important to acknowledge that stimuli used in reintroduction programmes to prepare animals for human conflict or predation, such as water pistols and predator models, have been criticized for producing unrealistic conditioned responses [94,175] and to date, their welfare outcomes have been neglected [176]. However, real-world alternatives such as live predators, harsh weather conditions or aggressive competitors are arguably a larger welfare concern that could lead to real pain and suffering or even fatality. Continued welfare debates must weigh whether causing an animal brief pain or suffering as part of a survival-relevant challenge is a necessary step towards a laudable, utilitarian conservation outcome to save a species at large [176].

With a challenge appraisal framework in hand for *ex-situ* populations, we can begin to move away from an *ex-situ* ‘captivity effect’ and towards an *ex-situ* ‘challenge effect’. This steers away from arbitrary classifications of captivity. The practicalities of this endeavour will not be covered in this paper because they require a far more collaborative approach with multiple stakeholders. But in brief, cognitive and behavioural records from live specimens could be synchronized to anatomical and neurological brain measurements (most likely post-mortem). Repositories of brain and skull material (e.g. primates [177]) could be paired with records of living specimen cognition and behaviour and the environmental challenges they have faced [178]. Thinking strategically, such an endeavour could be integrated into existing regional and international conservation programmes for endangered species, which already oversee the conservation breeding activities of many dozens of species in *ex-situ* sites worldwide [5,24,25].

### 5.2. Preserving behavioural and cognitive skills and flexibility

A challenge appraisal framework will identify inadequate challenges that need to be addressed. In other words, inadequate challenges need specific intervention to transform into adequate challenges. The ‘agony of choice’, i.e. the difficulty in choosing where to invest limited conservation funds in the current mass extinction crisis [179] is beyond the scope of this paper. However, there are species for which the most effective approach may be to use enrichment to develop a particular cognitive skill or more general behavioural or cognitive flexibility. More specifically, a cognitive enrichment approach centring around competence and agency [180] could prove very useful for reintroduction preparedness because it helps an animal become competent at a specific task, but also generalize to wider survival or breeding-relevant situations. As an example, zoo-housed chimpanzees (*Pan troglodytes*) who become competent at foraging from complex artificial termite mounds [181] might develop wider agency from this activity, as evidenced by accessing other cryptic food sources and using various tools to do so.

## 6. Conclusion

Ultimately, future research is needed to confirm whether actively preserving the ‘endangered brain’ *ex-situ* leads to better reintroduction outcomes than traditional approaches. This research must be interdisciplinary, to better understand how the environment and human interventions impact the animal brain, cognition and behaviour. However, this research will also feed back into specific research fields that focus on the proximate and ultimate causes of animal behaviour; from neuroscience and physiology to cognition. Coordination between *ex-situ* sites could accelerate the pace and impact of future research by encouraging its incorporation into species survival plans that focus efforts on particular taxonomic groups and regions. Hopefully, this review will ignite relevant conversations so that researchers and conservation managers can work collectively to evaluate and act upon any challenge effect in *ex-situ* populations.

## Data Availability

This article has no additional data.
